# Both complete response and long-term survival after combination therapy with toripalimab in a patient with meta-oligometastases cervical cancer: a case report

**DOI:** 10.3389/fimmu.2025.1542795

**Published:** 2025-04-07

**Authors:** Ge Jin, Jun Wang

**Affiliations:** ^1^ 1 Department of Gynecologic Oncology, Fourth Hospital of Hebei Medical University, Shijiazhuang, China; ^2^ Department of Radiation Oncology, Fourth Hospital of Hebei Medical University, Shijiazhuang, China

**Keywords:** cervical cancer, case report, long-term survival, radiotherapy, toripalimab

## Abstract

**Background:**

The therapeutic landscape for recurrent or metastatic cervical cancer remains limited, with few options available. According to National Comprehensive Cancer Network (NCCN) guidelines, pembrolizumab combined with chemotherapy, with or without bevacizumab, is recommended for affected patients. Despite these guidelines, recurrence rates remain elevated, and survival outcomes following standard interventions are unsatisfactory. Furthermore, real-world management of recurrent or metastatic cervical cancer presents inherent complexities, often requiring an integrative, multidimensional treatment approach to enhance long-term survival. The pressing need to refine and adopt multimodal therapeutic strategies is evident in addressing the persistent challenges associated with disease recurrence and progression.

**Case description:**

The case involved a 40-year-old female diagnosed with advanced cervical cancer who underwent radical hysterectomy. Postoperative pathology identified high-risk features, including lymph node involvement, necessitating adjuvant chemoradiotherapy. However, disease progression occurred during treatment, manifesting as metastases in the left supraclavicular and axillary lymph nodes. Subsequent local radiotherapy and systemic therapy led to a favorable response. By November 2024, overall survival (OS) had surpassed 72 months, with toripalimab administered for 65 months, during which no immunotherapy-related adverse events occurred.

**Conclusion:**

This case offers clinical insight into the efficacy and safety of integrating chemotherapy, immunotherapy, and radiotherapy in recurrent or metastatic cervical cancer. The multimodal approach contributes to prolonged survival in this patient. Further clinical trials are essential to substantiate the therapeutic benefits of this regimen in broader patient cohorts.

## Introduction

Cervical cancer (CC) ranks as the fourth most prevalent malignancy worldwide and remains a major contributor to cancer-related mortality among females, with approximately 604,000 new cases and 342,000 deaths reported in 2020 (1, 2). Despite a gradual increase in incidence, CC continues to impose a substantial global disease burden, second only to breast cancer. A negative correlation exists between CC burden and the socio-demographic index (SDI), with lower-income regions disproportionately affected due to insufficient public health resources (3). Once recurrence or metastasis occurs, prognosis is generally poor, particularly for those experiencing disease progression during treatment. Current guidelines from the National Comprehensive Cancer Network (NCCN) and the European Society of Gynecologic Oncology designate palliative chemotherapy as the standard of care for recurrent or metastatic cases (4, 5). However, its therapeutic efficacy remains suboptimal, as most patients derive limited clinical benefit, with only approximately one-fourth achieving a treatment response (6). The GOG-240 clinical trial demonstrated that the addition of bevacizumab to chemotherapy extended overall survival (OS) by 3.5 months, yielding modest improvements (7). Presently, pembrolizumab in combination with chemotherapy, with or without bevacizumab, is the recommended first-line treatment for recurrent or metastatic CC in patients with programmed death ligand 1 (PD-L1) positivity, defined by a combined positive score (CPS) of ≥1, as supported by findings from the KEYNOTE-826 study (8).

Lymph node metastasis (LNM) represents a significant independent prognostic determinant in CC, markedly affecting patient survival and serving as a primary conduit for tumor dissemination. Among metastatic sites, the parametrial and obturator foramen lymph nodes exhibit the highest incidence, followed by the internal, external, and common iliac nodes, as well as the para-aortic, supraclavicular, and infraclavicular lymph nodes. In contrast, axillary lymph node involvement remains uncommon. Existing research on LNM in CC predominantly centers on pelvic and para-aortic lymph nodes, with limited investigations addressing supraclavicular and axillary involvement. In recent oncological discourse, the classification of sync-oligometastases, metachronous oligometastases (meta-oligometastases), and oligo-recurrence has gained increased attention, emphasizing the prospect of prolonged survival or potential cure in patients with restricted metastatic burden when managed with appropriate local and systemic interventions.

Oligometastases are characterized by the presence of 1-5 metastatic or recurrent lesions, independent of the primary tumor’s status. Oligo-recurrence specifically denotes cases where 1-5 metastatic or recurrent lesions remain amendable to local therapy, provided the primary tumor is controlled, thereby representing a metachronous form of oligometastases. In contrast, sync-oligometastases involve concurrent metastatic disease with an active primary lesion. This report details a patient diagnosed with stage IIIC2p CC, initially treated with radical hysterectomy. During postoperative adjuvant radiotherapy, meta-oligometastases emerged, yet durable survival was achieved through a multimodal approach incorporating local radiotherapy, systemic chemotherapy, and immunotherapy.

## Case presentation

**Figure 1:**
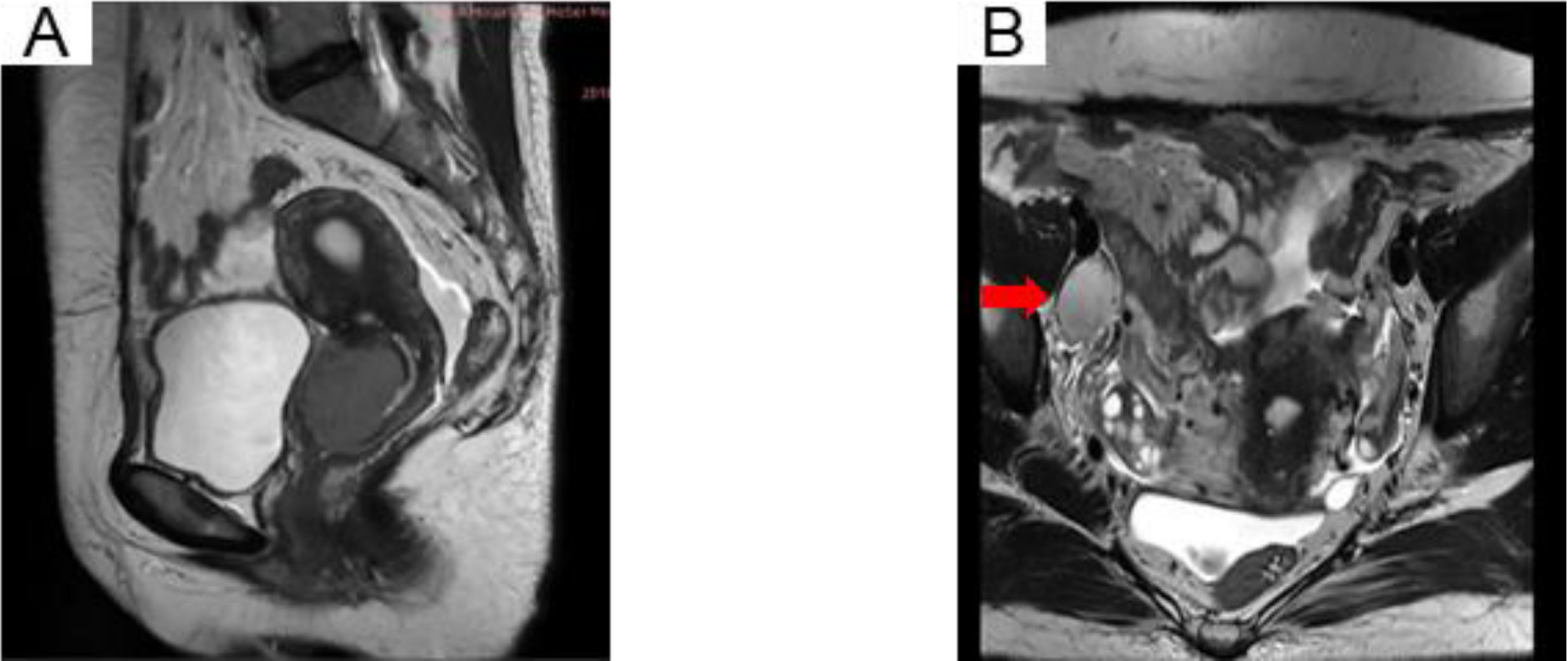
MRI pelvis showing an irregular uterine cervix soft tissue mass **(A)** and the right iliac vascular enlarged lymph node **(B)** in T2-weighted images.

The patient, a 40-year-old female, was admitted to the Fourth Hospital of Hebei Medical University on November 26, 2018, with a primary complaint of “contact bleeding for two weeks.” Obstetric history included four pregnancies, consisting of two induced abortions and two spontaneous deliveries. Physical examination identified no remarkable abnormalities, and her Eastern Cooperative Oncology Group (ECOG) performance status was assessed as 0. No underlying medical conditions, smoking history, or notable familial predispositions were reported. Pelvic magnetic resonance imaging (MRI) revealed a cervical mass with a maximum diameter of approximately 4 cm, alongside an enlarged right iliac vascular lymph node measuring 2.7 cm in short diameter (**Figure 1**). Chest X-ray showed no evident neoplastic lesions. The squamous cell carcinoma antigen (SCCA) level was elevated at 61.10 ng/ml. Based on the 2018 International Federation of Gynecology and Obstetrics (2018 FIGO) staging system, the diagnosis was classified as IIIC1r. Despite the NCCN guidelines recommending concurrent chemoradiotherapy, the patient declined treatment.

On November 30, 2018, the patient underwent transabdominal radical hysterectomy, bilateral salpingo-oophorectomy, and pelvic and para-aortic lymph node dissection under general anesthesia, performed by the gynecologic oncology team. Histopathological evaluation confirmed squamous cell carcinoma of the cervix, with a tumor dimension of 3.5 × 3.5 × 2.5 cm, exhibiting deep stromal invasion exceeding half of the cervical stroma and clear evidence of vascular infiltration. Lymph node analysis demonstrated extensive metastatic dissemination: (1) left pelvic region: metastases detected in one of seven lymph nodes; (2) right pelvic region: five of ten lymph nodes positive for metastases; (3) right common iliac region: five of seven lymph nodes involved; and (4) para-aortic region: all 16 lymph nodes exhibiting metastatic infiltration. Based on these pathological findings, the disease was classified as 2018 FIGO stage IIIC2p (**Table 1**).

**Table 1:** Summary of patient demographics.

Characteristics	Status
Age, years	40
Sex	Female
ECOG PS	0
Maximum tumor diameter (MR), cm	4.1
FIGO 2018 stage (postoperative)	IIIC2p
Local invasion	No
Nodal involvement	Pelvic, right common iliac, and para- aortic lymph nodes
Sites of metastases	No
Histological	Squamous cell cancer
HPV status	Unknown
Obstetric history	Four pregnancies, comprising two induced abortions and two spontaneous deliveries
Ethnicity	Chinese Han
Education	Unknown
Occupation	Housewife
Previous medical history	None
Tobacco smoking	No
Family history	None cancer-related history
Marital status	Married
Previous therapy	None

FIGO, International Federation of Gynecology and Obstetrics; ECOG PS, Eastern Cooperative Oncology Group (ECOG) performance status; MR, magnetic resonance; HPV, human papillomavirus.

**Figure 2:**
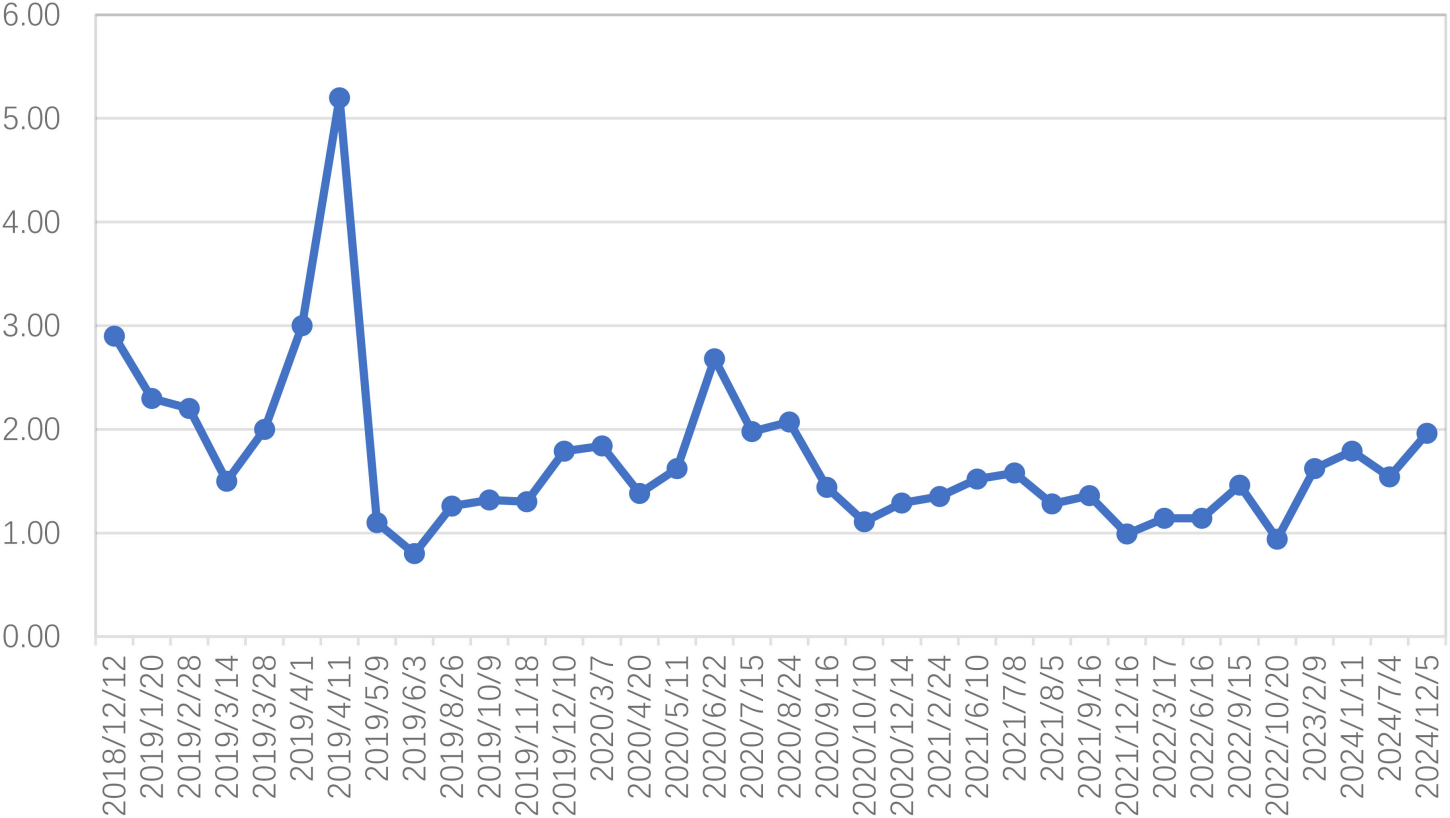
Squamous cell carcinoma antigen (SCCA) fluctuations from the period after radical hysterectomy to the last follow-up (reference value <1.5ng/ml before August 2019, reference value <2.7ng/ml after August 2019).

In alignment with NCCN guidelines, the patient’s high-risk profile for recurrence necessitated postoperative adjuvant therapy. The radiation oncology team implemented a regimen comprising three cycles of paclitaxel liposome (175 mg/m², d1) and lobaplatin (40 mg, d1) chemotherapy, administered every 21 days (q21d) from December 2018 to January 2019. Subsequently, intensity-modulated radiotherapy (IMRT) was employed to target both pelvic and extended para-aortic fields, delivering a cumulative dose of 48.6 Gy across 27 fractions beginning in January 2019. Due to the patient’s compromised physical status, concurrent chemotherapy was not pursued. Following the completion of external beam radiation, high-dose-rate brachytherapy with iridium-192 was introduced, delivering a prescribed dose of 7 Gy per fraction on a weekly basis for two fractions. SCCA levels were systematically monitored throughout the course of treatment (**Figure 2**).

**Figure 3:**
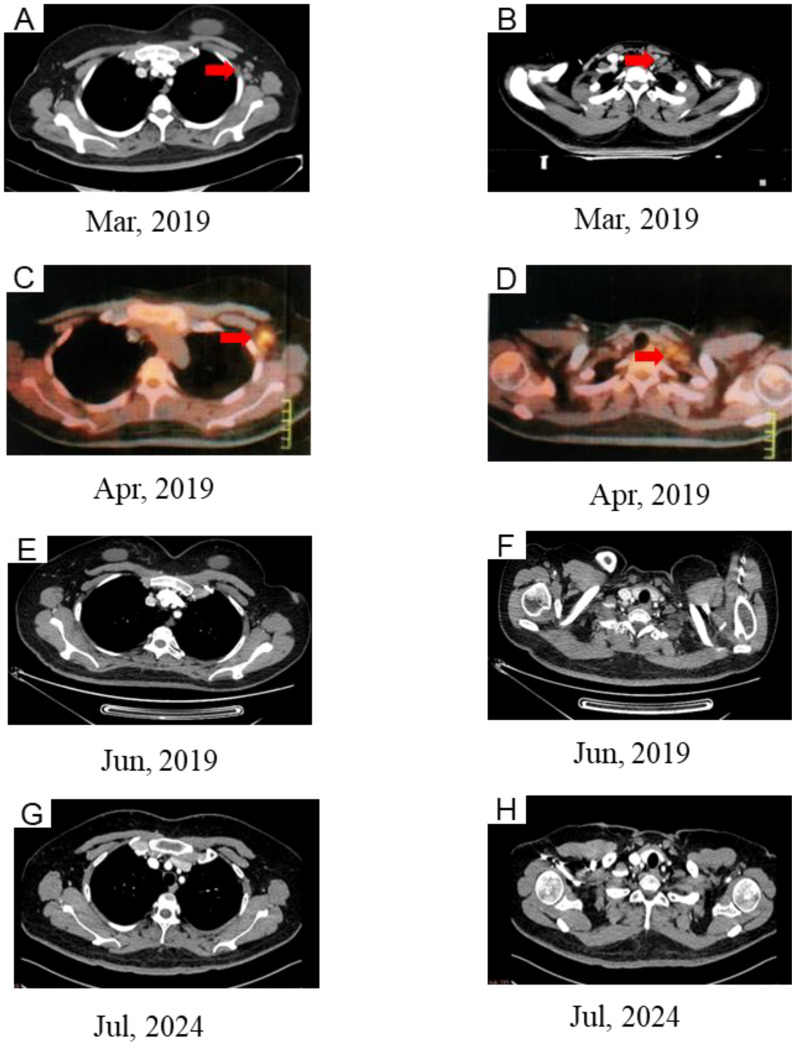
CT **(A, B)** and PET/CT **(C, D)** images showing new lesion in left supraclavicular and left axillary. **(E, F)** Complete response after chemoradiotherapy. **(G, H)** CT images at the last follow-up.

In February 2019, an SCCA level of 2.2 ng/ml was detected. Subsequent computed tomography (CT) imaging on March 29 identified mild enlargement of the left axillary lymph nodes, while no abnormalities were evident in the neck or lungs (**Figures 3A, B**). To further investigate, an ultrasound-guided biopsy of the left axillary lymph node was performed on April 3, confirming poorly differentiated carcinoma. However, immunohistochemical analysis was not performed due to the patient’s personal circumstances. To evaluate the extent of metastatic involvement, positron emission tomography-CT (PET-CT) imaging on April 4 demonstrated multiple enlarged lymph nodes exhibiting abnormal glucose hypermetabolism in the left posterior triangle, clavicular region, and axilla, indicating a high probability of LNM (**Figures 3C, D**).

On April 8, the patient received chemotherapy with the TP regimen, comprising albumin-bound paclitaxel (175 mg/m², d1) and cisplatin (50 mg/m², d2-3; q21d). Throughout treatment, SCCA levels remained abnormal, exhibiting a gradual rise from 2.2 to 5.2 ng/ml. IMRT was initiated on April 15, 2019, delivering a total dose of 50.4 Gy to the cervical region over 28 fractions at five sessions per week. Additionally, the supraclavicular and axillary metastatic lymph nodes received boost doses up to 60.2 Gy. However, severe bone marrow suppression and gastrointestinal toxicity rendered concurrent chemotherapy intolerable, leading to its discontinuation at the patient’s request. Subsequently, toripalimab, an immune checkpoint inhibitor (ICI), was introduced at 240 mg intravenously every three weeks. Complete remission was achieved by the end of treatment. At the 72-month follow-up, no evidence of recurrence or metastasis was detected (**Figures 3E-H**), and no immune-related adverse events (AEs) were observed.

## Discussion

Early-stage CC is associated with a favorable prognosis, with a five-year survival rate exceeding 90% for localized lesions. However, recurrence or metastasis significantly limits therapeutic options, leading to poor clinical outcomes. The five-year survival rate declines to approximately 17%, with a median survival duration of 8-13 months and OS ranging from 13 to 17 months (4, 9). In this case, stage IIIC2p CC was diagnosed following standard radical hysterectomy. According to NCCN guidelines, patients presenting with high-risk factors post-hysterectomy require concurrent chemoradiotherapy (CCRT) with cisplatin-based regimens. Notably, the STARS study, a randomized clinical trial conducted in China, indicated that sequential chemoradiation (SCRT) yielded superior disease-free survival (DFS) rates and a lower risk of cancer-related mortality compared to radiotherapy alone in females with CC (10).

Following this clinical trial, the patient completed three cycles of TP chemotherapy before undergoing IMRT. However, concurrent chemotherapy was withheld during radiotherapy due to myelosuppression. Despite postoperative adjuvant therapy, supraclavicular and axillary LNM emerged. Research suggests that even after pelvic lymph node dissection, 10-15% of patients initially classified as node-negative (N0) may later develop lymphatic recurrence or metastasis (11). Although less frequent in advanced CC, hematogenous metastasis remains a concern, with the lungs (36.3%), bones (16.3%), liver, and brain identified as common metastatic sites (12). A study by Jung Ho Im et al. compared treatment outcomes between patients with distant LNM and those with visceral organ metastases, demonstrating significantly improved progression-free survival (PFS) and OS in cases of isolated distant LNM (13). Informed by these observations, a proactive strategy was implemented to address the patient’s disease progression.

In clinical practice, palliative chemotherapy remains a primary therapeutic approach for recurrent or metastatic CC. However, in this case, severe chemotherapy-related toxicity limited the patient to a single course of the TP regimen. Prospective studies have investigated the efficacy of PD-1 inhibitors in this patient population, reporting an objective response rate (ORR) ranging from 12.2% to 55.6% (13–16). Based on this evidence, pembrolizumab received approval from the United States Food and Drug Administration (FDA) for PD-L1-positive, locally advanced CC progressing during or after chemotherapy.

The introduction of immunotherapeutic agents has markedly reshaped the treatment paradigm for recurrent or metastatic CC, a historically challenging malignancy. However, real-world treatment complexities persist. In 2019, the high cost and restricted accessibility of pembrolizumab imposed considerable limitations on its clinical use. Toripalimab, a recombinant humanized IgG4 monoclonal antibody, serves as an alternative by inhibiting PD-1 interactions with its ligands, PD-L1 and PD-L2. Its therapeutic potential has been observed across multiple malignancies, including melanoma, urothelial carcinoma, renal cell carcinoma, nasopharyngeal carcinoma, and other solid tumors (17–20). A study involving 24 patients assessed the efficacy and safety of toripalimab in combination with bevacizumab and platinum-based chemotherapy as a first-line regimen for refractory recurrent or metastatic CC. With a median follow-up of 18.6 months (range, 3.3-28.5), the ORR reached 83.3%, while the disease control rate (DCR) was 95.8%. Among participants, 9 (37.5%) achieved a complete response, 11 (45.8%) showed a partial response, and 3 (12.5%) maintained stable disease. The median PFS was 22.6 months, whereas the median OS remained unreached (21). Additionally, in an exploratory trial assessing the efficacy of ICIs in combination with CCRT for locally advanced cervical cancer, the addition of the anti-PD-1 agent toripalimab exhibited a favorable safety profile and achieved an ORR of 100%, with PD-L1 expression on tumor cells deemed non-essential for eligibility (22). In this case, following pre-immunotherapy assessments, toripalimab treatment commenced in May 2019 at a dosage of 240 mg every three weeks. Notably, no immune-related AEs were observed throughout the treatment course. Despite disease progression, including supraclavicular and axillary LNM during postoperative adjuvant therapy, the patient obtained exceptional long-term survival exceeding five years. This outcome, attributed to the integration of immunotherapy, chemotherapy, and radiotherapy in managing metastatic lesions, marks a substantial advancement in immunotherapeutic approaches, achieving results previously considered unattainable. However, the substantial role of radiotherapy in this success must not be overlooked. A comprehensive, multimodal therapeutic strategy may offer more sustained benefits for complex and refractory malignancies such as recurrent or metastatic CC.

Survival outcomes following radiotherapy for patients with supraclavicular LNM have shown considerable variation. Matthew S et al. evaluated 38 patients undergoing definitive irradiation to oligometastatic sites of CC, reporting a median OS of 50.7 months from the completion of irradiation, with 2- and 3-year OS rates of 74% and 65%, respectively. Median PFS reached 21.7 months, with 1- and 2-year PFS rates of 63% and 48% (23). Another study reported 3- and 5-year OS rates of 75.0% and 56.3%, alongside PFS rates of 66.7% and 41.7% for the same intervals (24). Cao et al. analyzed 60 patients with stage IVB CC presenting with oligometastases at initial diagnosis, demonstrating superior PFS and OS outcomes in those receiving definitive irradiation to primary and oligometastatic sites compared to chemotherapy combined with pembrolizumab, with or without bevacizumab (25). Variability in survival rates across studies may be attributed to differences in sample size, prescription dose, and radiotherapy techniques. In this case, radiotherapy was integral to managing distant metastases, with the patient receiving 60.2 Gy in 28 fractions via IMRT at five fractions per week. Lee et al. reported effective control of supraclavicular LNM with prescription doses below 66 Gy, though their analysis was constrained by a limited sample size of seven patients (24). Another study identified an association between doses below 50 Gy and reduced PFS, advocating for a minimum dose of 50 Gy to enhance disease control (26). Cao et al. administered radiation doses between 60 and 74 Gy to metastatic lymph nodes, demonstrating favorable outcomes in metastasis management (25).

In the immunotherapy era, radiotherapy enhances treatment efficacy by enhancing antigen presentation, increasing T-cell infiltration, and reshaping the immune microenvironment. When integrated with ICIs, radiotherapy intensifies immune activation, leading to improved clinical outcomes (27–31). The KEYNOTE-A18 trial, a randomized, double-blind, placebo-controlled phase 3 study, demonstrated that pembrolizumab combined with chemoradiotherapy significantly prolonged OS in patients with LACC, establishing this regimen as the new standard of care (32). Similarly, a meta-analysis indicated that CCRT with ICIs led to superior PFS and OS compared to CCRT alone. The combination group exhibited higher ORR than the control group, while grade ≥3 treatment-related AEs occurred at comparable rates. However, immunotherapy-related AEs of any grade were more frequently observed in the combination cohort (33).

In this case, the patient received immunotherapy and local radiotherapy but, due to personal medical considerations, did not undergo systemic chemotherapy. Despite this, a complete response was achieved, with sustained long-term survival free from treatment-related or immune-related AEs. Such outcomes remain uncommon yet promising, emphasizing the therapeutic potential of integrated treatment strategies. Further investigation through prospective randomized controlled trials is warranted to refine and validate the efficacy of radiotherapy and immunotherapy combinations in recurrent or metastatic CC.

## Conclusions

The therapeutic strategy in this case aligns with evidence-based protocols in recurrent or metastatic CC management, integrating data from clinical trials. This approach plays a key role in achieving prolonged survival. Additionally, the case offers clinically relevant evidence on the effectiveness and safety of combining immunotherapy, chemotherapy, and radiotherapy in treating recurrent or metastatic CC.

Given recent advancements in the management of recurrent or metastatic CC, several key questions arise:

### How should radiotherapy be chosen for oligometastatic lesions?

Patients with oligometastases, particularly those with lymph node involvement, generally exhibit a comparatively favorable prognosis. However, uncertainty remains regarding the optimal approach for local irradiation, specifically in choosing between conventional fractionated and hypofractionated radiotherapy. The KROG 14-11 study assessed local control and survival outcomes in patients with recurrent or oligometastatic uterine CC treated with stereotactic body radiotherapy (SBRT) using CyberKnife. A total of 85 patients received a median dose of 39 Gy in three fractions, corresponding to a biologically effective dose (BED) of 90 Gy, with toxicity levels remaining within acceptable limits. Local PFS rates at 2 and 5 years were 82.5% and 78.8%, respectively (34). Additionally, a phase II study involving 50 patients with recurrent gynecologic cancer and single or multiple (≤4) metastases reported a 96% target response rate (48 of 50 patients) following robotic-assisted CyberKnife SBRT (24 Gy in three daily fractions). Furthermore, 68% (34 of 50 patients) achieved clinical benefit for at least six months (95% CI, 53.2–80.1), with no subsequent need for systemic therapy due to disease progression (35). In comparison, Cao et al. employed IMRT or volumetric modulated arc therapy (VMAT) for oligometastatic lymph node irradiation, an approach consistent with the one applied in the present case (25). The radiation oncologist managing the oligometastatic case utilized 3D-printed templates for interstitial brachytherapy targeting inguinal LNM, delivering a prescription dose of 25 Gy (36). Additionally, large-scale analyses, including those derived from the Surveillance, Epidemiology, and End Results (SEER) database, indicate that radiotherapy may contribute to improved survival in metastatic CC. Careful patient selection for radiotherapy remains essential, considering prior treatments, lesion distribution, and overall health status (37). In clinical practice, treatment strategies should be tailored to individual patient profiles to optimize therapeutic outcomes.

### Optimal combination of radiotherapy and immunotherapy

What is the optimal combination model for radiotherapy and immunotherapy in recurrent or metastatic CC?

Radiotherapy remains a cornerstone in the management of locally advanced CC. For patients with 2018 FIGO stage IB3-IVA, cisplatin-based chemoradiotherapy followed by image-guided adaptive brachytherapy (IGABT) is established as the gold standard (38). The integration of CCRT with ICIs has been shown to enhance immune activation, increasing both central and effector memory T-cell populations, indicative of immune modulation (39). Multiple studies have investigated the role of ICIs in LACC, demonstrating their antitumor potential. The KEYNOTE-A18 randomized, double-blind, phase 3 trial, for instance, revealed that the addition of pembrolizumab to CCRT significantly improved PFS and reduced mortality by 33% in LACC patients (32), broadening the role of immunotherapy in CC and influencing clinical practice. Moreover, clinical evidence supports the efficacy of combining immunotherapy with chemotherapy, with or without bevacizumab, in recurrent or metastatic CC (40, 41). However, key uncertainties remain: Can the integration of immunotherapy with chemoradiotherapy, with or without bevacizumab, yield additional benefits for patients with oligometastatic CC? Is sustained immunological maintenance necessary after achieving disease control? Addressing these uncertainties requires large-scale, high-quality clinical trials to refine treatment strategies.

### What are the treatment options for recurrent metastatic cervical cancer ≥2 line?

With ICIs now integrated into frontline therapy, subsequent disease relapse presents limited treatment alternatives. Tissue factor, a critical component of the blood coagulation cascade, has been identified as a transmembrane protein involved in the proliferation and invasion of certain cancer cells, positioning it as a potential therapeutic target (42–44). The Innovate Tisotumab Vedotin 301 (innovaTV 301) trial recently demonstrated the efficacy and safety profile of tisotumab vedotin (2.0 mg/kg) compared with physician-selected chemotherapy in 502 patients (median overall survival: 11.5 months vs. 9.5 months; hazard ratio for death from any cause: 0.70; 95% CI, 0.54–0.89; *P* = 0.004), leading to FDA approval on April 29, 2024. Notably, the drug’s effectiveness was independent of tissue factor expression in tumor cells (45).

HER2 expression was observed in approximately 5% of all CC cases (46). Trastuzumab deruxtecan (T-DXd), a HER2-directed ADC, has been evaluated for CC treatment within the phase II DESTINY-PanTumor02 basket trial (47). This study assessed T-DXd at 5.4 mg/kg every three weeks across seven cohorts comprising patients with various HER2-positive (IHC 2-3+) advanced solid tumors that had progressed following at least one prior systemic therapy, with ORR as the primary endpoint. Between 2020 and 2022, 267 patients received treatment, including 40 with CC. Among the CC cohort, 6 of the 8 women achieved an objective response.

The STING signaling pathway has garnered significant interest in tumor immunotherapy, particularly in combination with immune checkpoint inhibitors, emerging as a focal point in clinical research (48). By detecting DNA damage signals in tumor cells, such as cytoplasmic DNA accumulation, STING activation triggers the secretion of type I interferons (IFN-I) and chemokines, promoting CD8+ T cell and NK cell infiltration into the tumor microenvironment. This process facilitates the transition from an immunologically “cold” to “hot” tumor phenotype, thereby enhancing ICI efficacy. MK-1454, a structurally modified CDNs STING agonist developed by Merck, exhibits high affinity for STING and, when administered via intratumoral injection, induces complete tumor regression. Its combination with pembrolizumab in patients with advanced solid tumors or lymphoma has demonstrated a favorable safety profile and improved therapeutic efficacy. Currently, MK-1454 is being evaluated in a Phase II clinical trial for its efficacy and safety in head and neck squamous cell carcinoma (NCT04220866). The STING agonist MSA-2 functions as a non-covalent dimer binding to STING. Both oral and subcutaneous administration of MSA-2 have demonstrated safety and tolerability in mouse models, exhibiting sustained anti-tumor immunoefficacy either as monotherapy or in combination with PD-1 inhibitors (49). Despite significant advancements in research on the STING signaling pathway and its modulators, several challenges remain, primarily concerning (1) potential adverse reactions and (2) limited clinical efficacy.

## Data Availability

The original contributions presented in the study are included in the article/supplementary files. Further inquiries can be directed to the corresponding author.
